# Antiphospholipid Syndrome and Antibodies Associated With Malignancy and Older Age: A Retrospective Study

**DOI:** 10.7759/cureus.59891

**Published:** 2024-05-08

**Authors:** Michael Liu, Kim Griffin, Kaavya Nair, Nikita Chhabra, Ehab Harahsheh, Adnan Shahid, Eugene Scharf

**Affiliations:** 1 Neurology, Mayo Clinic, Rochester, USA; 2 Internal Medicine, Mayo Clinic, Rochester, USA; 3 Neurology, Mayo Clinic, Scottsdale, USA

**Keywords:** hypercoagulability, malignancy, antiphospholipid antibodies, antiphospholipid syndrome, stroke

## Abstract

Background: Antiphospholipid syndrome (APLS) is an established cause of thrombosis and hypercoagulability. However, the clinical characteristics of those with APLS or patients with positive antiphospholipid antibodies (APLA) in the embolic stroke of undetermined source (ESUS) have not been well studied.

Methods: A retrospective analysis was conducted between January 1, 2010, and December 31, 2020, across all three Mayo Clinic sites. Patients who were included in the study were tested for APLA and had a diagnosis of ESUS. Baseline characteristics, radiographic parameters, and outcome data were collected and compared between those who tested positive for APLS or had positive APLA and those who were negative.

Results: A total of 206 patients were included in the study. Eight (4%) patients were diagnosed with APLS, and 21 (10%) patients had positive APLA. On comparing those with a diagnosis of APLS and those without, patients with APLS were found to be significantly older (75 years old ± 9 vs. 58 years old ± 14, p = 0.001) and were more likely to have a history of cancer (50% vs. 13%, p = 0.012). Those with positive APLA had similar findings of being older (67 years old ±13 vs. 58 years old ± 14 p = 0.003) and more likely to have a history of cancer (29% vs. 8.4% p = 0.027). Radiographically, those with APLS had a higher white matter disease burden (Fazekas score median 2 (IQR 1.5-3) vs. median 1 (IQR 1-2), p = 0.028).

Conclusion: Both APLS and positive APLA are associated with older age and a history of malignancy. These findings highlight the importance of considering a hypercoagulable evaluation even in the elderly ESUS population.

## Introduction

Stroke is a leading cause of morbidity and mortality in the United States, with the majority of these strokes being ischemic in nature. Stroke affects approximately 795,000 people per year, with 610,000 of those being diagnosed with a first stroke [1]. Investigating stroke etiology is critical to preventing recurrent cerebrovascular events. An estimated 30% to 40% of strokes are classified as cryptogenic in nature [2]. Embolic stroke of undetermined source (ESUS) is a subcategory of cryptogenic stroke and is defined as a non-lacunar-appearing stroke on imaging with no significant atherosclerotic lesion in the territory of the stroke and no other causative factor noted [3]. Much research has gone into studying the ESUS construct and unmasking the etiology of these strokes. Part of this evaluation includes looking at hypercoagulable states such as antiphospholipid syndrome (APLS).

Antiphospholipid syndrome is an autoimmune disorder that is associated with thrombosis and hypercoagulability in the presence of antiphospholipid antibodies (APLA) [4,5]. It is thought to predominantly affect younger individuals, especially younger women, though epidemiologic data is mixed and varies between cohorts [6,7]. Some evidence also supports its association with malignancy [8-10]. The neurologic manifestations of APLS are diverse and are thought to have a relationship with recurrent ischemic stroke. However, these studies have been inconsistent, and criticisms arise from the heterogeneity of the cohorts [5,11-14]. Specifically, APLS has not been well studied in the ESUS cohort.

As clinical practice trends toward testing for APLS and other hypercoagulable states in those who are younger and with recurrent thrombotic events in the ESUS cohort, further study is necessary to understand the characteristics of APLS and the presence of APLA in the ESUS cohort [15,16]. Our study aims to better characterize the clinical characteristics as well as evaluate those at risk of testing positive for APLA and a subsequent diagnosis of APLS in those suffering from ESUS.

## Materials and methods

Participants

Consecutive patients ≥ 18 years old who presented to any Mayo Clinic site (Rochester, Jacksonville, or Arizona in the USA) with a diagnosis of ESUS between January 1, 2010, and December 31, 2020, were screened for this retrospective study. The label of ESUS was given to patients based on previously established definitions and was determined by board-certified neurologists [3]. Patients included in our study must have received at least one measure of serum APLA testing. Antiphospholipid antibody testing included anti-cardiolipin IgM, anti-cardiolipin IgG, beta-2-glycoprotein 1 IgM, beta-2-glycoprotein IgG, and lupus anticoagulant. Testing for serum APLAs was done at the discretion of the expert physician taking care of the patient. Patients lacking serum APLA testing were excluded from our study. Patient follow-up was updated until the study end date of December 31, 2020.

Endpoints

The primary endpoints of our study included the development of APLS or positive APLAs. The diagnosis of APLS was made by the patient’s treating board-certified physician per the Sapporo classification criteria [4]. Positive APLAs were determined by our clinical laboratory cutoffs (Mayo Clinic Laboratories, Rochester, MN, USA) and are set at the following: anti-cardiolipin IgM (≥ 15.0 MPL), anti-cardiolipin IgG (≥ 15.0 GPL), beta-2-glycoprotein 1 IgM (≥ 15.0 MPL), and beta-2-glycoprotein IgG (≥ 15.0 GPL). Lupus anticoagulant presence was evaluated through the Mayo Clinic Laboratories lupus anticoagulant profile testing algorithm after evaluating prothrombin time, activated partial thromboplastin time, and dilute Russell viper venom time (see Appendix A). We aimed to evaluate factors that predicted the primary endpoints. Factors that were evaluated included demographic information, patient characteristics, cardiac evaluation, laboratory testing, and radiographical features. Secondary endpoints were also evaluated and included clinical outcomes such as the development of recurrent stroke, new malignancy, new venous thromboembolism (VTE), and new atrial fibrillation.

Statistical analysis

Statistical analysis was completed using the assistance of SPSS Statistics version 27 (IBM Corp., Armonk, NY, USA). The analysis of means between groups was completed using independent sample T-tests. Chi-square or Fisher exact tests were used to analyze categorical variables between two groups. The Mann-Whitney U test was used for comparison between medians across groups. The significance for these analyses was set at p<0.05. Univariate and multivariate logistic regression were completed to identify predictors of our endpoints. Significant variables in univariate logistic regression were established at p≤0.1. Only significant variables in univariate logistic regression were included in the multivariate analysis, with significance set at p≤0.05. 

## Results

Demographics and clinical history

During the study period, 206 patients met the inclusion criteria. Of those patients, the average age at their ESUS event was 59 ± 14 years, 112 (54%) were male, 196 (95%) were Caucasian, and 91 (44%) had a history of smoking. Medical comorbidities included 124 (60%) patients with hypertension, 118 (57%) with hyperlipidemia, 58 (28%) with obstructive sleep apnea (OSA), 43 (21%) with diabetes, 32 (16%) with coronary artery disease, 25 (12%) with a history of autoimmune disease, 58 (28%) with a history of stroke or TIA, 27 (13%) with a history of cancer, and 18 (9%) with a history of VTE. Before presentation, 68 (33%) patients were on antithrombotics. Regarding acute therapies, 17 (8%) received IV thrombolysis alone, seven (3%) received mechanical thrombectomy alone, and 11 (5%) received a combination of mechanical thrombectomy and thrombolysis. For secondary stroke prevention, 107 (52%) patients were started on a single antiplatelet agent post-stroke, 64 (31%) on dual antiplatelet therapy, 24 (12%) on anticoagulation, 11 (5%) on combination anticoagulation and antiplatelet (Table 1).

**Table 1:** Clinical characteristics and outcomes APLA: Antiphospholipid antibodies, APLS: Antiphospholipid syndrome, VTE: Venous thromboembolism

Variables	All patients (n = 206)	APLA positive (n = 21)	p-value	APLS diagnosed (n = 8)	p-value
Clinical characteristics					
Age, years, mean (SD)	59 (14)	67 (13)	0.003	75 (9)	0.001
Female, n (%)	94 (46)	11 (52)	0.512	3 (38)	0.473
Caucasian, n (%)	196 (95)	21 (100)	0.879	8 (100)	0.980
History of smoking, n (%)	91 (44)	17 (81)	0.224	3 (38)	0.499
Hypertension, n (%)	124 (60)	14 (67)	0.523	7 (88)	0.149
Hyperlipidemia, n (%)	118 (57)	12 (57)	0.989	6 (75)	0.471
Diabetes, n (%)	43 (21)	4 (19)	1.000	2 (25)	0.673
Obstructive sleep apnea, n (%)	58 (28)	4 (19)	0.445	2 (25)	1.000
Coronary artery disease, n (%)	32 (16)	3 (14)	1.000	2 (25)	0.360
History of stroke/transient ischemic attack, n (%)	58 (28)	5 (24)	0.686	2 (25)	0.580
History of autoimmune disease, n(%)	25 (12)	3 (14)	0.726	2 (25)	0.251
History of cancer, n(%)	27 (13)	6 (29)	0.027	4 (50)	0.012
History of VTE, n(%)	18 (9)	2 (10)	1.000	1 (13)	0.525
Previous antithrombotic, n (%)	68 (33)	8 (38)	0.790	4 (50)	0.527
Acute stroke therapeutics, n (%)					
Thrombolysis alone	17 (8)	1 (5)		0 (0)	
Thrombectomy alone	7 (3)	0 (0)	0.422	0 (0)	0.636
Combination	11 (5)	0 (0)		0 (0)	
Post-stroke antithrombotic, n(%)					
Single antiplatelet	107 (52)	14 (67)		5 (63)	
Dual antiplatelet	64 (31)	7 (33)	0.173	3 (38)	0.636
Anticoagulation	24 (12)	0 (0)		0 (0)	
Combination	11 (5)	0 (0)		0 (0)	
Antiphospholipid antibodies					
Anticardiolipin IgM, MPL, mean (SD) n=180	11 (13)	34 (8).	0.055	34 (48)	0.193
Anticardiolipin IgG, GPL, mean (SD) n=181	11 (15)	42 (9)	0.097	48 (66)	0.168
Beta-2-glycoprotein IgM, MPL, mean (SD) n=176	11 (16)	44 (10)	0.060	62 (23)	0.130
Beta-2-glycoprotein IgG, GPL, mean (SD) n=177	12 (19)	51 (11)	0.014	64 (23)	0.021
Lupus anticoagulant, n (%), n=193	7 (4)	7 (33)	<0.001	4 (50)	<0.001
Radiographic features					
Baseline white matter burden (Fazekas scale) median score (IQR) n=194	1 (1-2)	1 (1-3)	0.108	2 (1.5-3)	0.028
Cortical infarct, n (%)	189 (92)	17 (81)	0.079	7 (88)	0.656
Stroke location					
Anterior circulation only, n (%)	125 (61)	10 (48)		3 (38)	
Posterior circulation only, n (%)	58 (28)	10 (48)	0.097	4 (50)	0.339
Both anterior and posterior, n (%)	23 (11)	1 (5)		1 (13)	
Multiple territories, n (%)	38 (18)	2 (10)	0.379	2 (25)	0.642
Clinical outcomes					
Recurrent stroke, n (%)	46 (22)	5 (24)	0.864	2 (25)	0.853
New VTE, n (%)	14 (7)	1 (5)	1.000	1 (13)	0.436
New cancer diagnosis, n (%)	4 (2)	0 (0)	1.000	0 (0)	1.000
New atrial fibrillation, n (%)	12 (6)	2 (21)	0.351	0 (0)	1.000
Follow-up, days, mean (SD)	788 (2966)	344 (359)	0.471	255 (181)	0.605

Cardiac evaluation

Of the 206 patients included in the study, 205 had some type of echocardiography done as part of their evaluation. Of these, 154 (75%) had a transesophageal echocardiogram completed, and 51 (25%) only had a transthoracic echocardiogram completed. Eighty-four (41%) patients had a patent foramen ovale (PFO) present, 11 (5%) had an atrial septal aneurysm, and 48 (23%) were noted to have an enlarged left atrium on evaluation. Left ventricular aneurysm was seen in four (2%). Of these patients, 171 patients received prolonged cardiac monitoring; for one (0.5%) patient, the duration of monitoring was unknown; 13 (8%) received a 24-hour Holter monitor, 34 (20%) received a 48-hour Holter monitor, 76 (44%) received a 30-day Holter monitor, and 47 (27%) received an implantable loop recorder as their longest form of cardiac monitoring (Table 2).

**Table 2:** Cardiac characteristics PFO: Patent foramen ovale

Variables	All patients (n = 205)	APLA positive (n = 21)	p-value	APLS diagnosed (n = 8)	p-value
Transesophageal echocardiogram, n (%)	154 (75)	15 (71)	0.790	7 (88)	0.682
PFO, n (%)	84 (41)	9 (21)	0.853	4 (50)	0.719
Enlarged left atrium, n (%)	48 (23)	9 (43)	0.026	4 (50)	0.089
Left ventricular aneurysm, n (%)	4 (2)	0 (0)	1.000	0 (0)	1.000
Atrial septal aneurysm, n (%)	11 (5)	1 (5)	1.000	0 (0)	1.000
Prolonged Cardiac Monitoring (n = 171)					
Unknown duration	1 (0.5)	0 (0)		0 (0)	
24-hour monitor	13 (8)	0 (0)		0 (0)	
48-hour monitor	34 (20)	4 (19)	0.562	2 (25)	0.910
30-day monitor	76 (44)	6 (29)		3 (38)	
Implantable loop recorder	47 (27)	6 (29)		2 (25)	

Antiphospholipid laboratory evaluation

All 206 patients received APLS testing. Of these, 180 (87%) received anti-cardiolipin IgM testing (mean 11 MPL ± 13), 181 (88%) received anti-cardiolipin IgG testing (mean 11 GPL ± 15), 176 (85%) received beta-2-glycoprotein IgM testing (mean 11 MPL ± 16), and 177 (86%) received beta-2-glycoprotein IgG testing (mean 12 GPL ± 19). Lupus anticoagulant was tested in 193 (94%) patients and was positive in seven (4%). Twenty-one patients had at least one aspect of their APLA test positive. The average anti-cardiolipin IgM was 24 MPL ± 34, anti-cardiolipin IgG was 25 GPL ± 42, anti-beta-2 glycoprotein IgM was 28 MPL ± 44, and anti-beta-2 glycoprotein was 40 GPL ± 51. 7 (4%). Of these patients, eight were diagnosed with APLS with an average anti-cardiolipin IgM testing of 34 MPL ± 48, anti-cardiolipin IgG of 48 GPL ± 66, anti-beta-2 glycoprotein IgM of 48 GPL ± 63, and anti-beta-2 glycoprotein IgG of 77 GPL ± 65 (Table 1).

Radiographic features

A total of 189 (92%) patients were found to have either a cerebral or cerebellar cortical infarct. Seventeen (8%) had a hemorrhagic transformation of their stroke, 28 (14%) had strokes bilaterally, 125 (61%) patients had strokes in the anterior circulation, 58 (28%) in the posterior circulation, and 23 (11%) in both. And 38 (18%) patients had infarctions in multiple territories. The baseline white matter burden as measured by the Fazekas scale was a median of 1 (IQR 1-2) (Table 1).

Outcomes

The average follow-up time was 788 days ± 2966. Forty-six (22%) patients were found to have recurrent stroke, 14 (7%) were found to have a new VTE, four (2%) had a new diagnosis of cancer, and 12 (6%) had a new diagnosis of atrial fibrillation. Eight (4%) patients were diagnosed with APLS formally, while 21 (10%) tested positive for APLA.

APLS and APLA-positive patients

Eight patients were diagnosed with APLS. When evaluating the differences between the APLS-diagnosed patients and those without, those diagnosed with APLS were significantly older (75 years old ± 9 vs. 58 years old ± 14, p = 0.001) and were more likely to have a history of cancer (50% vs. 13%, p = 0.012). The median baseline white matter disease burden was higher (Fazekas score median 2 (IQR 1.5-3) vs. median 1 (IQR 1-2)). There were no other differences between other demographic, clinical, cardiac, radiographic features, follow-up time, or outcomes (Tables 1-2). On univariate regression analysis, age (p = 0.003, OR 1.14, 95% CI 1.04-1.24), history of cancer (p = 0.006, OR 7.609, 95% CI 1.78-32.52), enlarged left atrial size (p = 0.087, OR 3.477, 95% CI 0.84-14.47), and white matter disease burden (p = 0.028, OR 2.414, 95% CI 1.10-5.31) were significant. On multivariate regression analysis, only age was significant (p = 0.014, OR 1.12, 95% CI 1.02-1.23) (Table 3).

**Table 3:** Regression analysis of those diagnosed with APLS APLS: Antiphospholipid syndrome, VTE: Venous thromboembolism, PFO: Patent foramen ovale

Variables (n = 206)	p-value	Odds ratio	95% confidence interval
Age	0.003	1.138	1.044-1.239
Gender	0.338	0.490	0.114-2.106
Ethnicity			
Black	1.000	1.000	0
Asian	1.000	1.000	0
Caucasian	0.999	68740000	0
Hispanic	1.000	1.000	0
Other	1.000	N/A	N/A
Smoking			
Active	0.998	0	0
Former	0.881	1.119	0.258-4.845
Never	0.989	N/A	N/A
Hypertension	0.143	4.846	0.585-40.147
Hyperlipidemia	0.314	2.304	0.454-11.696
Diabetes	0.770	1.276	0.248-6.559
Obstructive sleep apnea	0.840	0.845	0.166-4.314
Coronary artery disease	0.458	1.867	0.360-9.688
History of stroke/transient ischemic attack			
Silent infarct	0.998	0	0
Yes	0.719	1.352	0.262-6.989
No	0.937	N/A	N/A
History of autoimmune disease			
Suspected	0.999	0	0
Yes	0.287	2.464	0.469-12.938
No	0.567	N/A	N/A
History of cancer	0.006	7.609	1.780-32.517
History of VTE	0.703	1.521	0.177-13.103
Enlarged left atrium (n = 205)	0.087	3.477	0.836-14.471
PFO (n = 205)	0.598	1.462	0.355-6.019
Left ventricular aneurysm (n = 205)	0.999	0	0
Atrial septal aneurysm (n = 205)	0.999	0	0
Cortical infarct	0.659	0.615	0.071-5.319
Anterior/posterior circulation			
Anterior	0.369	N/A	N/A
Posterior	0.158	3.012	0.652-13.923
Both	0.602	1.848	0.184-18.590
Multiple territories	0.628	1.500	0.291-7.737
White matter disease burden (Fazekas score) (n = 194)	0.028	2.414	1.098-5.305
Recurrent stroke	0.853	1.167	0.227-5.984
New VTE	0.522	2.033	0.232-17.795
New cancer	0.999	0	0
New atrial fibrillation	0.999	0	0
Multivariate analysis (n = 206)			
Age	0.024	1.116	1.014-1.228
Enlarged left atrium	0.360	2.100	0.428-10.303
History of cancer	0.164	3.055	0.633-14.748
White matter burden (Fazekas score)	0.621	1.269	0.494-3.264

Twenty-one patients tested positive for APLA. Seven patients were positive for anticardiolipin IgM, five patients for anticardiolipin IgG, five for beta 2 glycoprotein IgM, nine patients for beta 2 glycoprotein IgG, and seven patients for lupus anticoagulant. Evaluating the differences between those with APLA positivity versus those without, those with positive APLA were significantly older (67 years old ±13 vs. 58 years old ± 14 p = 0.003), more likely to have a history of cancer (29% vs. 8.4% p = 0.027), and more likely to have enlarged left atria (43% vs. 23% p = 0.026). No other differences were seen (Tables 1-2). On univariate regression analysis, age (p = 0.005, OR 1.06, 95% CI 1.02-1.10), history of cancer (p = 0.034, OR 3.12, 95% CI 1.09-8.93), enlarged left atrial size (p = 0.031, OR 2.79, 95% CI 1.10-7.09), cortical infarct (p = 0.070, OR 0.321, 95% CI 0.094-1.095), white matter disease burden (p = 0.062, OR 1.62, 95% CI 0.98-2.70), and posterior location of stroke (p = 0.068, OR 2.40, 95% CI 0.937-6.127) were significant. On multivariate regression analysis, posterior location (p = 0.024, OR 3.76, 95% CI 1.23-11.45), anterior location (p = 0.025; but not both anterior and posterior circulation), and enlarged left atria (p = 0.050, OR 1.41, 95% CI 0.74-2.67) were significant (Table 4).

**Table 4:** Regression analysis of those diagnosed with positive APLA antibodies APLA: Antiphospholipid antibodies, VTE: Venous thromboembolism, PFO: Patent foramen ovale

Variables (n = 206)	p-value	Odds ratio	95% confidence interval
Age	0.005	1.055	1.016-1.095
Gender	0.513	0.740	0.300-1.827
Ethnicity			
Black	1.000	1.000	0
Asian	1.000	1.000	0
Caucasian	0.999	19380000	0
Hispanic	1.000	1.000	0
Other	1.000	N/A	N/A
Smoking			
Active	0.243	2.140	0.597-7.672
Former	0.111	2.271	0.829-6.221
Never	0.237	N/A	N/A
Hypertension	0.524	1.364	0.525-3.539
Hyperlipidemia	0.989	0.994	0.399-2.474
Diabetes	0.828	0.881	0.280-2.768
Obstructive sleep apnea	0.333	0.571	0.184-1.775
Coronary artery disease	0.868	0.897	0.248-3.240
History of stroke/transient ischemic attack			
Silent infarct	0.403	0.413	0.052-3.283
Yes	1.000	1.000	0.313-3.190
No	0.702	N/A	N/A
History of autoimmune disease			
Suspected	0.999	0	0
Yes	0.787	1.197	0.326-4.397
No	0.964	N/A	N/A
History of cancer	0.034	3.124	1.093-8.928
History of VTE	0.893	1.112	0.237-5.210
Enlarged left atrium (n = 205)	0.031	2.788	1.096-7.094
PFO (n = 205)	0.853	1.090	0.437-2.716
Left ventricular aneurysm (n = 205)	0.999	0	0
Atrial septal aneurysm (n = 205)	0.897	0.870	0.106-7.155
Cortical infarct	0.070	0.321	0.094-1/095
Anterior/posterior circulation			
Anterior	0.114	N/A	N/A
Posterior	0.068	2.396	0.937-6.127
Both	0.546	0.523	0.064-4.293
Multiple territories	0.278	0.436	0.097-1.956
White matter disease burden (Fazekas score) n=194	0.062	1.623	0.976-2.699
Recurrent stroke	0.864	1.098	0.379-3.176
New VTE	0.698	0.662	0.082-5.327
New cancer	0.999	0	0
New atrial fibrillation	0.451	1.842	0.376-9.036
Multivariate analysis (n = 206)			
Age	0.104	1.041	0.992-1.092
History of cancer	0.405	1.762	0.465-6.670
Enlarged left atrium	0.050	2.864	1.001
Cortical infarct	0.094	0.307	0.077-1.224
Anterior/posterior circulation			
Anterior	0.025	N/A	N/A
Posterior	0.020	3.758	1.234-11.448
Both	0.299	0.305	0.032-2.865
White matter burden (Fazekas score)	0.299	1.405	0.739-2.672

Of note, when looking at clinical outcomes, those with APLA positivity did not have a higher risk of recurrent stroke over the observed follow-up period (24% vs. 22%, p = 0.789), new VTE (4.8% vs. 7%, p = 1.00), or a new diagnosis of cancer (0% vs. 2.2%, p = 1.00). Similarly, with patients diagnosed with APLS, there were no significant differences in rates of recurrent stroke (25% vs. 22%, p = 1.00), new VTE (13% vs. 6.6%, p = 0.436), or new diagnosis of cancer (0% vs. 2.0%, p = 1.00). Follow-up times were also not significantly different between those with APLA positivity (344 days ± 359 vs. 838 days ± 3125, p = 0.471) and those with a diagnosis of APLS (255 days ± 181 vs. 809 days ± 3024, p = 0.605) (Table 1).

## Discussion

In the exploratory study of 206 patients with ESUS who had APLA testing, 21 (10.2%) had at least one positive phospholipid antibody. Eight (3.8%) patients were diagnosed with APLS. Those who tested positive for APLA or were diagnosed with APLS were more likely to be older and have a history of cancer. White matter disease burden was associated with the diagnosis of APLS but not the presence of APLA antibodies. The enlarged left atrial size was associated with the presence of APLA but not the diagnosis of APLS. On multivariate regression analysis, older age was the strongest predictor of the diagnosis of APLS and enlarged left atrial size was the strongest predictor of positive APLA. Our study highlights a broader consideration of APLS in older patients presenting with ESUS, especially if they have a history of cancer.

Antiphospholipid syndrome has often been thought of as a disease of the young, with an estimated age of onset around 50 years old, and is often thought to have a higher predisposition in female patients, which may be due to the association between APLS and systemic lupus erythematosus (SLE) [6,7,17,18]. The selected retrospective cohort highlights that this may not be the case, with the mean age of those testing positive for APLA being 67 years old and the mean age of those diagnosed with APLS being 75 years old. There were also no significant differences in gender in our cohort, which has also been demonstrated in other instances [7]. Cancer has a suspected association with APLS, and the development of APLA and APLS at an older age in our cohort may be driven by the association seen with malignancy [19,20]. We speculate that a history of malignancy may contribute to residual thrombosis risk, possibly through the APLA pathway. The overall frequency of APLAs in the general population is also poorly understood, and the estimated prevalence is less than 5%, whereas our cohort was double that, likely attributable to the selection of our patients, but this does highlight the higher than expected APLA-positive patients in the ESUS cohort [6].

Our study also shows the increased prevalence of white matter disease burden in these patients. This relationship has been previously described and is associated with cognitive impairment as well. Though the true mechanism is unknown, it is postulated that the microvasculature is susceptible to thrombotic events leading to these changes. While not directly examined in the present study, there does not seem to be a relationship with larger vessel arterial disease burden, such as carotid stenosis, as previously suggested in the literature [5,21-23].

The significance of the observed relationship between an enlarged left atrium and the presence of APLA is unclear in this cohort. This relationship has not previously been reported in the literature and may reflect confounding factors given the older age reported in the APLA group. Reports of cardiac manifestations in APLS primarily focus on cardiac valvular lesions as well as accelerated atherosclerotic deposition [24-26]. It can be postulated that both of these cardiac manifestations may lead to enlargement of the left atria through downstream effects, but this is purely hypothetical. Furthermore, this relationship was not seen with those diagnosed with APLS in our study.

The clinical impact of APLA antibody testing and APLS positivity was not shown to be significantly different in our cohort. Specifically, there were no differences in rates of recurrent stroke or vascular thrombosis in our population. The risk of vascular thrombosis is well documented and is part of the diagnostic criteria for APLS [4]. It is difficult to say why our results differ, but it could be related to either the treatment effect or our study being retrospective and not designed to detect a difference in this clinical endpoint. The clinical relevance of APLA positivity without a diagnosis of APLS is still a question, especially as it pertains to IgM antibodies, which may often be transient and of unclear clinical significance. Similar results have been seen in a large meta-analysis looking at recurrent stroke risk in patients with APLA positivity. This meta-analysis may have been confounded by non-standard cutoff values as well as different types of APLA tested and variable inclusion criteria [12]. The question of whether APLS in different populations is significant and requires different treatment modifications has yet to be fully understood.

Our study has several limitations. The retrospective nature of this study presents a limitation. The single health system aspect of this study limits its generalizability. The lack of set uniform criteria for when to test patients for APLA, secondary to the retrospective nature of this study, does allow for bias that limits the generalizability of this study. Additionally, few clinical outcomes were achieved, likely due to the small cohort of APLA-positive patients or patients diagnosed with APLS, the need for further prolonged follow-up, and treatment modification effects. Further studies in prospectively collected cohorts or a randomized clinical trial would yield more definitive conclusions.

## Conclusions

Our study investigates the relationship between clinical characteristics and APLA positivity or the diagnosis of APLS in ESUS patients. The presence of APLS might be considered more broadly in ESUS patients who are older and have a history of malignancy. The clinical significance of APLA is unclear in recurrent strokes, yet it may change clinical management if detected.

## Figures and Tables

**Figure 1:**
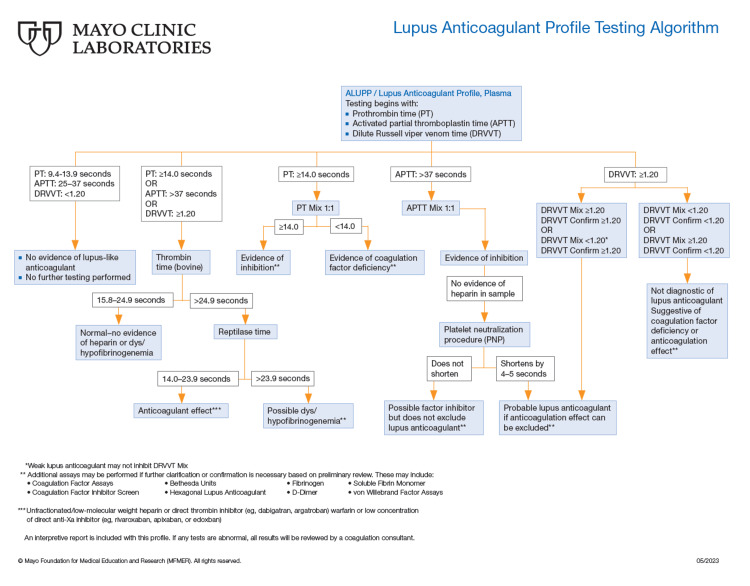
Lupus anticoagulant profile testing algorithm

## Appendices

Appendix A

The Mayo Clinic Laboratories lupus anticoagulant profile testing algorithm is displayed in Figure 1.
